# Legitimation problems of participatory processes in technology assessment and technology policy

**DOI:** 10.1007/s10202-012-0123-4

**Published:** 2012-11-16

**Authors:** Thomas Saretzki

**Affiliations:** Center for the Study of Democracy, Leuphana University Lueneburg, Scharnhorststrasse 1, 21335 Lüneburg, Germany

## Abstract

Since James Carroll (1971) made a strong case for “participatory technology”, scientists, engineers, policy-makers and the public at large have seen quite a number of different approaches to design and implement *participatory processes* in technology assessment and technology policy. As these participatory experiments and practices spread over the last two decades, one could easily get the impression that participation turned from a theoretical normative claim to a working practice that goes without saying. Looking beyond the well-known forerunners and considering the ambivalent experiences that have been made under different conditions in various places, however, the “if” and “how” of participation are still contested issues when questions of technology are on the agenda. *Legitimation problems* indicate that attempts to justify participation in a given case have not been entirely successful in the eyes of relevant groups among the sponsors, participants, organizers or observers. Legitimation problems of participatory processes in technology assessment and technology policy vary considerably, and they do so not only with the two domains and the ways of their interrelation or the specific features of the participatory processes. If we ask whether or not participation is seen as problematic in technology assessment and technology policy-making and in what sense it is being evaluated as problematic, then we find that the answer depends also on the approaches and criteria that have been used to legitimize or delegitimize the call for a specific design of participation.

## Abstract (de)

Seit James Carroll (1971) für das Konzept einer “participatory technology” plädierte, konnten Wissenschaftler, Ingenieure, Politiker und die Öffentlichkeit eine Vielzahl von unterschiedlichen Ansätzen im Bereich des Designs und der Umsetzung von *partizipativen Prozessen* in der Technologiefolgenabschätzung (TA) und in der Technologiepolitik beobachten. Angesichts der Ausbreitung dieser partizipatorischen Experimente und Praktiken in den letzten zwei Jahrzehnten konnte man leicht den Eindruck gewinnen, dass Partizipation nun keine bloß theoretisch begründete normative Forderung mehr darstellt, sondern zu einem selbstverständlichen Bestandteil der Praxis geworden ist. Jenseits der bekannten Vorreiter zeigen die ambivalenten Erfahrungen, die an verschiedenen Orten unter unterschiedlichen Bedingungen mit den Ansätzen einer partizipativen TA gemacht worden sind, jedoch, dass sowohl das “Ob” als auch das “Wie” von Partizipation weiterhin umstritten sind, wenn Technologien auf der Agenda stehen. *Legitimationsprobleme* verweisen darauf, dass Versuche zur Rechtfertigung von Partizipation in einem bestimmten Fall nicht oder doch nicht gänzlich erfolgreich waren, jedenfalls nicht in den Augen wichtiger Gruppen unter den Trägern, Beteiligten, Organisatoren oder Beobachtern eines solchen Prozesses. Fragt man nach Legitimationsproblemen von partizipatorischen Prozessen in der TA und der Technologiepolitik, so zeigen sich bedeutende Unterschiede und diese Unterschiede hängen nicht nur mit den beiden unterschiedlichen Bereichen der TA und der Technologiepolitik und der jeweiligen Art und Weise ihres Wechselverhältnisses zusammen, sondern auch mit den spezifischen Merkmalen der jeweils in Rede stehenden partizipatorischen Prozesse. Wenn danach gefragt wird, ob und in welchem Sinn Partizipation in der TA oder Technologiepolitik in einem bestimmten Fall als problematisch bewertet wird oder nicht, dann hängt die Antwort schließlich auch davon ab, welcher Ansatz und welche Kriterien herangezogen wurden, um die Forderung nach Partizipation und ihre spezifische Ausgestaltung zu legitimieren oder zu delegitimieren.

## Résumé

Depuis que James Carroll plaida (en 1971) pour l’idée d’une “participatory technology”, scientifiques, ingénieurs, politiciens et le public assistèrent à une multitude d’approches différentes de conception et de mise en œuvre de *processus participatifs* dans l’évaluation des conséquences technologiques et la politique technologique. En voyant les expériences et pratiques participatives des deux décennies écoulées, on pouvait facilement avoir l’impression que la participation n’était plus une exigence normative théorique mais qu’elle était devenue un automatisme. Toutefois, au-delà des pionniers connus, les expériences ambivalentes acquises dans les approches d’une évaluation participative des conséquences technologiques à différents endroits dans des conditions individuelles montrent que la controverse persiste sur le “si” et le “comment” de la participation lorsque des technologies sont en jeu. *Des problèmes de légitimation* indiquent que des tentatives de justifier la participation dans un certain cas n’ont pas ou seulement en partie mené à bien, au moins à l’avis de groupes influents parmi les sponsors, participants, organisateurs ou observateurs d’un tel processus. Les problèmes de légitimation de processus participatifs dans l’évaluation des conséquences technologiques et de la politique technologique diffèrent de manière considérable, ces différences n’émanant pas seulement des deux domaines différents de l’évaluation et de la politique et de leur corrélation, mais aussi du caractère spécifique de chacun des processus participatifs en question. Lorsque l’on se demande si la participation dans l’évaluation des conséquences technologiques ou la politique technologique est considérée comme problématique ou pas dans un certain cas, la réponse dépendra en fin de compte aussi de l’approche et des critères ayant été pris en compte pour légitimer ou délégitimer l’exigence de participation et sa conception spécifique.

## Introduction

Since political scientist James Carroll (1971) made a strong case for “participatory technology” in the leading scientific journal “Science” 40 years ago, scientists, engineers, policy-makers and the public at large have seen quite a number of different approaches to design and implement participatory processes in technology assessment and technology policy. As these participatory experiments and practices spread over the last two decades and some forms became almost standard operating procedures in some Western democracies, one could easily get the impression that participation turned from a theoretical normative claim to a working practice that goes without saying. Looking beyond the well-known forerunners and considering the ambivalent experiences that have been made with different designs under different conditions in various places, we recognize that evaluations of “participatory technology” still differ. It is still a contested issue whether or not participation is the way to go when questions of technology are on the agenda and if so, how participatory processes should be designed and implemented.

The task of this paper is to reconsider this debate about participation with a special focus on the question of legitimacy from the perspective of a political scientist. To that end, I shall start with a short recollection of the idea of “participatory technology” and first evaluations of the experiences made with the implementation of participatory approaches related to technology focussing on problems of legitimacy (2). While the original idea of “participatory technology” as well as the title of the international conference at the Institute of Technology Assessment (ITA) in Vienna related the issue of participation to “questions of technology” in general, a focus on legitimacy suggests the need to distinguish more clearly between technology assessment and technology policy. Both these fields are different object domains with different functions (3). Having introduced these distinctions with regard to the subject area, the next basic step is to say something about the concepts of legitimacy and legitimation from a political science perspective in order to clarify in what sense we can understand the notion of “legitimate hope” as one pole of the alternative presented at the conference in Vienna (4). Reconsidering the role of participation in technology assessment and policy then requires rethinking the reasons that have been given to justify it in the first place: Why should there be participation when it comes to “questions of technology”? (5) Looking at the opposite pole of the juxtaposition, the suggestion that participation in technology assessment might be nothing more than an “illusion” calls for some kind of comparison with the available “evidence”: What do we know about experiences with that “illusion” from empirical studies and evaluations of processes and practices that claim to be participatory? (6) On the grounds of some reflections about the theoretical concepts involved, about the ways the claim for participation has been justified with regard to technology assessment and policy and how that claim has been implemented in empirically observable participatory processes, I can then turn to the question of legitimation problems of these participatory processes (7) and look at specific problems these processes imply for the role of TA-experts who act as policy analysts in these participatory settings (8). The paper ends with a summary and conclusion about what appears to be the politics of legitimation and delegitimation with regard to participatory approaches in technology assessment and policy (9).

## Participatory technology: from emerging trends to early experiences in the 1970s

Political science has a rather long history of analysing, recommending and evaluating participation related to “questions of technology”. The wave of democratization at the end of the 1960s provided the background for an increasing interest in these issues beyond the boundaries of the academic discipline. A prominent early piece is an article entitled “participatory technology” that political scientist James Carroll (1971) published in the leading scientific journal “Science” in the early 1970s. Addressing an audience that consists mainly of natural scientists, Carroll (1971: 647) did not begin with normative considerations but started to “analyze several facts of which people are becoming increasingly aware that suggest why participatory technology is emerging as a trend”. Like other scholars at the time, he interpreted the call for more participation as a reaction to social impacts of technology which would lead to alienation. And he explained the next step that was leading from the sociological diagnosis of social alienation to the political claim for (more) participation which was accompanied by various questions of legitimation.

There is considerable speculative and observational evidence … that the scope and complexities of science and technology are contributing to the development of social alienation in contemporary society. …I analyze the incipient emergence of participatory technology as a countervailing force to technological alienation in contemporary society. I interpret participatory technology as one limited aspect of a more general search for ways of making technology more responsive to the felt needs of the individual and of society. The term *participatory technology* refers to the inclusion of people in the social and technical processes of developing, implementing, and regulating a technology, directly and through agents under their control, when the people included assert that their interests will be substantially affected by the technology and when they advance a claim to a legitimate and substantial participatory role in its development and implementation. (Carroll 1971: 647)

Carroll’s short formula “participatory technology” suggests a broader focus on technology and a concern of drawing the attention to the political dimensions inherent in technological development as such. His explanation entails a line of reasoning that was further developed by philosophers of technology (e.g. Winner 1980) to justify the normative claim to a “legitimate and substantial participatory role” when “questions of technology” are on the agenda. That line of reasoning draws an analogy of constructing and implementing technologies to processes of law making. In this view, technology is no longer seen as “neutral” or as an instrument that could be used for all kinds of purposes, but as value-loaded and with the power to enforce these values and norms.

One primary reason for the emergence of participatory technology is the realization that technology often embodies and expresses political value choices that, in their operations and effects, are binding on individuals and groups, whether such choices have been made in political forums or elsewhere. … To an indeterminate extent, technological processes in contemporary society have become the equivalent of a form of law – that is, an authoritative or binding expression of social norms and values from which the individual or a group may have no immediate recourse. (Carroll 1971: 648)

As a political scientist, Carroll (1971: 649) did not only point to new rights and opportunities participation might bring with regard to a more open deliberation. He also stressed the limits of such an approach when it comes to binding decisions and their implementation. New participatory processes might include, enable and empower more participants. But in the context of a modern constitutional democracy, these processes would not put the participants outside of the law.

Participatory technology is one limited way of raising questions about the specific technological forms in terms of which social change is brought about. It is directed toward the development of processes and forums that are consistent with the expectations and values of the participatory individuals, who may resort to them in the absence of other means of making their views known. In participatory technology, however, as in other participatory processes, the opportunity to be heard is not synonomous (sic!) with the right to be obeyed. (Carroll 1971: 649)

What appeared as an emerging trend in the beginning of the 1970s turned into an experimental, but at the same time controversial praxis that could already be analysed and evaluated by social scientists at the end of the decade. In the beginning, problems of legitimacy were associated with technologies and the established non-participatory forms of technological policy-making. Now the reforms invented to solve the problems which lead to the call for more participation appeared to have legitimation problems of their own. In a prominent article, Dorothy Nelkin and Michael Pollak (1979: 55) highlighted the strategic interaction of organized groups of citizens demanding participation and governments offering participatory settings to overcome the legitimation problems of existing technology policies which were including only experts and administrations. In this perspective, participation appears not only as a demand from below, but also as a strategic move from above. After almost 10 years of experience, what researchers observed looked like an arena where citizens and non-governmental groups were interacting with governmental agencies in something that could be described as the politics of legitimation and delegitimation in various fields related to “questions of technology”.

‘Demystification’, ‘accountability’, ‘citizen participation’. These are slogans of recent disputes in the U.S. and Western Europe through which citizens’ groups have sought a voice in technological decisionmaking. … Moreover, the disputes have challenged the legitimacy of the technical experts and authorities heretofore responsible – almost exclusively – for decisions that are technologically based but which have dramatic social impacts. Awareness of the decline in public trust has stimulated a great variety of government efforts to involve citizens more directly in creating and implementing policies on technology. (Nelkin and Pollak 1979: 55)

Nelkin and Pollak (1979) examined these efforts under a heading that does not differ too much from the title of the conference in Vienna 30 years later. Their evaluation of “Public participation in technological decisions” was structured by the alternative: “Reality or grand illusion?” In their cross-country review, Nelkin and Pollak (1979: 55) found that the procedures invented by governments as reforms showed considerable variations with regard to their understanding of public participation and the role of technical and political information in the decision-making processes.

But most such participatory ‘reforms’ have been based on the assumption that they will lead to the acceptance of controversial technologies and to the restoration of the legitimacy of decision-making institutions. The procedures considered appropriate depend on national political styles and on the perceived nature of the problem of public acceptability. If lack of confidence is thought to be a problem arising from insufficient technical evidence, then the goal is to ascertain ‘scientific truth’. This leads to a structure based on scientific advice to public representatives. If the controversy is defined in terms of alienation, a more participatory or consultative system is developed. And if the problem of public consensus is defined in terms of inadequate information, it is assumed that people oppose technologies because they are poorly informed. The task then becomes one of ‘education’. (Nelkin and Pollak 1979: 55)

According to Nelkin and Pollak, these participatory reforms have their own legitimation problems, as they presuppose a limited and one-sided definition of what the original problem of legitimacy was and how it could be solved:

Most of these procedures rest on a traditional ‘welfare model’ – in which risks are defined as problems to be dealt with, mainly by experts. It is assumed that if a problem is solved by a respected group of elites or by using the best available scientific opinion, this will enhance the legitimacy of public authorities. And it is assumed that adequate information will contribute to consensus. Yet conflict and mistrust persist, and the procedures themselves are often debunked. Neither public participation nor enlightened representation appear to assure the acceptability of controversial technology. (Nelkin and Pollak 1979 60)

To overcome the persistent mistrust and conflicts, Nelkin and Pollak suggest an approach that does not rest on a one-sided definition of the legitimation problems involved, but takes the questions of opposing citizens seriously to proceed with a mutually recognized definition of problems and controversies.

What then must one do to enhance legitimacy? What kind of procedures would be acceptable to critical groups? To explore the sources of persistent cynicism about procedural reforms, we turn to five questions frequently asked by opposition groups: How are the boundaries of the problem defined? Who participates in the experiment? Who conducts the procedure? What is the distribution of technical expertise? Is there really a choice? (Nelkin and Pollak 1979: 60)

Today, almost all problem-solving strategies are confronted with an idea that originated and gained currency in contexts dominated by management sciences: the idea that at the end of “evidence-based” comparative research, policy analysts should come up with a proposal which can be suggested as “best practice” and then transferred to other contexts. Interestingly enough, this idea already played a role in structuring expectations 30 years ago. However, Nelkin and Pollak already did not see any meaningful way of meeting this expectation when problems of legitimation are at stake. Quite to the contrary:

Comparative policy studies often approach common problems, seeking a ‘best solution’ that can be transferred to other contexts. Our analysis is not to be interpreted in this way. The structure of experiments, and the assumptions about who should participate, reflect basic political differences and cannot be simply transferred without considerable adjustment. Indeed, transferring means of conflict resolution can pose problems not unlike those of technology transfer. (Nelkin and Pollak 1979: 64)

Yet, this rejection of the idea of “best solutions” does not imply that nothing generalizable can be learned from the experiments in participatory experiments related to technology policy.

What can be generalized is not the structure of the experiments, but the conditions that will allow dissenting groups to express their concerns and to communicate effectively with administrative agencies. These conditions include: A ‘formula’ that gives due weight to social and political factors; appropriate involvement of affected interests; an unbiased management; a fair distribution of expertise; and a real margin of choice. Actually, such procedural conditions are not likely to produce consensus, but they may reduce public mistrust and hostility toward political and administrative institutions in order to allow détente. Our conclusion, in fact, is that détente is a more appropriate and realistic goal. (Nelkin and Pollak 1979: 64)

Reconsidering the findings and evaluations of Nelkin and Pollak (1979) at the end of the 1970s in relation to some of the more recent critiques of participatory approaches (see below), one might very well get the feeling of a déjà vu, as if some problems and issues associated with participation have a tendency to be forgotten over the years and need to be (re-)discovered in every new wave of democratization after a while. Certainly the early 1990s have seen another wave of democratization after the downfall of the iron curtain. And recent developments in Germany suggest that after the first decade of the twenty-first century, yet another citizen protest culture is on the rise questioning established policy-making processes in the name of more participation and transparency. Against this background, one might get the impression that participation is now generally to be considered as established practice that goes without saying and needs no justification. However, such an impression is misleading if we analyse issues related to science and technology.

## “Questions of technology”, technology assessment and technology policy

What precisely is the object area we are talking about when we relate technology to the idea of participation? In a certain rather basic empirical sense, one could argue that the term “participatory technology” is something like a truism. Technologies do not construct, invent or distribute themselves in modern societies by their own force or nature (although some of its promoters and opponents sometimes create that impression). There is always someone involved or taking part in these societal processes of developing technologies (technologization) or applying specific physical techniques or using technical artefacts (technization). Thus, one might say that there is always some kind of participation of some actors in these processes related to technology. Scientists, engineers and industrialists are usually the first groups of actors that people in modern societies will name if asked to say who the actors are playing the most important roles in processes of inventing und distributing new technologies. If translated into such a framework that is not entirely technocentric in the sense of being conceptually devoid of any human actors, then the idea of “participatory technology” is more than a truism insofar as it raises the question: Who is or should participate in what kind of activity related to technology? And since there was an established set of actors who were always already involved in these activities at the time, the idea of participatory technology came to mean primarily going beyond the established actor constellation comprising interested innovators of a technological community and related office-holders in public administrations. And it meant raising the question: Who else is or should be participating in the development, use and regulation of a particular technology? In the context of these debates, processes would be called “participatory” if they would include actors who are not office-holders in the established institutions or are not already entitled to take part in the assessment procedures of the scientific and political system (Saretzki 2003: 44–46).

While the TA 2011 conference as a whole defines its object area broadly as “questions of technology”, the perspective taken here will not address issues of participation that might arise in all societal fields which are or could be subject to processes of technologization or technization. I shall rather focus on technology assessment and technology policy as two different, but strongly interrelated domains. The claim is, on the one hand, that issues of participation are and should be considered differently in each of these domains with regard to problems of legitimation. Since these domains are structured by different institutional principles and fulfil different functions, problems of legitimation arise and are discussed on different grounds. While both have to do with “technology” as their subject matter, the basic explicit function of TA is to provide some kind of “assessment”, whereas the activities associated with the domain of technology policy go beyond the level of knowledge production and assessment and proceed to the level of making and implementing binding decisions. On the other hand, as these domains are strongly interrelated in many policy processes, I argue that specific problems of legitimation in particular cases cannot be understood adequately without analysing and evaluating also the specific ways of their interrelation in concrete contexts. From an overall perspective, we can see that participatory designs have been coupled (or decoupled) with one or both of these domains—a difference that makes a difference with regard to its possible impacts.

*Technology policy* (TP) can be understood as a functionally and institutionally differentiated policy field. It is part of the public order or more precisely: part of the political system and includes all deliberations and decisions in the political system explicitly and intentionally related to processes of technologization and technization. As for their different functions, technology policies may be distributive by offering financial support for some technologies, redistributive by transferring resources from one field of technology to another, regulatory by defining the rules and regulations or even constitutive by establishing the principles for a technological regime. Since decisions of the political system are binding for its members, it is generally understood in a democracy that these members as citizens have a right to participate in the decision-making process that leads to decisions which they finally all have to accept as binding. Of course, the ways citizens can actually exercise these rights depend on the way their democratic institutions are designed and functioning. And—to pick up one point in Carroll’s (1971: 647) definition of “participatory technology”—it makes a difference, whether they can do it “directly” (i.e. in an institutional setting that corresponds with models of direct or participatory democracy) or “through agents under their control” (which corresponds with models of representative democracy). Yet in principle, within a policy field which clearly is part of a democratic political system, the logic of this system suggests that it should not be the “if” but the “how” that may give rise to controversies over the legitimacy of participation in technology policy.

*Technology assessment* (TA) can be conceived of as a functionally and institutionally differentiated system of (technology-related) knowledge production and evaluation. Its most cited specific function is to produce and evaluate knowledge about the preconditions of inventing, options of shaping and consequences of applying (new) technologies in society and the environment and to formulate alternative options for policies related to these technologies. Historically, some prominent institutions of technology assessment (like the OTA) have been initiated and sponsored by parts of the political system, assigned primarily with a narrow function of advising and counselling those political decision-makers who work within the established institutions like parliament or administration on specific issues on the public agenda of technology policy. Public controversies about complex and contested technologies play an important role in these institutionalization processes. While some TA institutions became part of the legislative or administrative branch of government, others grew out of civil society activities or developed out of problem-oriented research within the established scientific system.

**Fig. 1:**
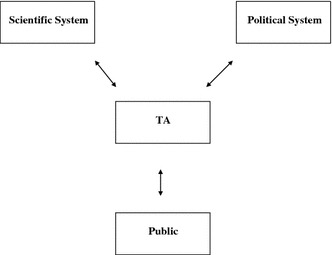
Technology assessment mediating between science, politics and the public

Today, in many industrialized democracies, TA institutions form some kind of network which can be conceptualized in a simplified model as a system mediating between the political-administrative system, the scientific system and the public (Fig. 1).

According to this concept, neither TA is part of the scientific system nor does it entirely belong to the political system. If it is also oriented towards the public sphere, TA has a mission of its own which makes it an advocate of enlightened understanding on the side of all three addressees: What do office-holders, scientists and citizens have to know to make reflective judgements and enlightened decisions on issues of technology? Conceptualizing TA in such a way as mediating between two institutionally and functionally differentiated systems and the public without being part of one of them has a number of implications. It implies that TA has to fulfil complex communicative functions that are not appropriately captured if we understand TA as a set of instruments from a tool box or as a social technology (to assess physical technologies). Instrumentalist, tool box or technological models of TA fail to address its specific communicative and mediating tasks. With regard to their communicative functions, it is more appropriate to conceptualize TA as an analytic-deliberative process mediating between the functionally and institutionally differentiated systems of science and politics and the public (Stern and Fineberg 1996; Saretzki 2005: 354–355).

To make a distinction between technology assessment and technology policy provides the conceptual framework for a differentiated consideration of the theory and practice of participation in these fields. Participatory processes can be observed (or called for) in technology assessment (pTA) or in technology policy (pTP) or in both domains. The standard operating procedure in both domains is still “non-participatory” (in the sense of not going beyond the conventional arrangements of established scientific experts and political elites to include other actors, especially citizens). Most of the debates on the role of participation relate to cases where we observe participation in TA, for example, citizens or stakeholders being involved in producing some kind of assessment and recommendations for technology policy (see Table 1). The output of these pTA processes (e.g. citizen reports) can then be taken up as an input to deliberations, decision-making and implementation within the established political arenas and institutions. Thus, participation of citizens or stakeholders in TA does not necessarily include their participation in the field of technology policy.

**Table 1:** Participation in technology assessment and technology policy

	Non-participatory technology policy	Participatory technology policy
Non-participatory technology assessment		
Participatory technology assessment		

Nonetheless, some proponents as well as some opponents of pTA are assuming that pTA also implies a participatory mode of technology policy-making in the sense of inventing a policy field fully in accordance with the model of a participatory democracy. This assumption is neglecting the difference between the domains of technology assessment and technology policy. To modify the remark James Carroll (1971: 649) made very early in the debate: the right to take part in the formulation of problem definitions and policy proposals is not the same as the right to take part in decision-making binding for the citizenry.

## “Legitimate hope” or “mere illusion”? Concepts of legitimacy and legitimation in political science

At first glance, the notion of “legitimate hope” which was presented as one pole at the conference in Vienna is somewhat puzzling from a political science perspective. It puts together two terms—“legitimation” and “hope”—which many political scientists would rather understand as belonging to two different spheres. Why? To find an answer, let us look at the meaning of the terms. To start with the second: What is hope? According to standard dictionaries of current English, a first meaning of hope is that of a “feeling of expectation and desire; feeling of trust or confidence” (Hornby et al. 1972: 476). If hope is a feeling, can hopes (i.e. feelings) be qualified as legitimate or illegitimate? Although there is some rethinking of the role of emotions in political science in general and policy inquiry in particular (Fischer 2009: 272–294), the concept of legitimation as understood in political science is still primarily related to processes of justification by giving reasons. If hope is understood as a feeling or emotion, hope can certainly be a strong motif, and its frustration can be deeply demotivating, especially for those who shared that feeling in the beginning. But what is its relation to reason giving, which requires relating it to a system of reference that provides the grounds for its legitimacy? Other explications from more sophisticated dictionaries describe the meaning of hope as an “expectation” or “prospect” of something to come (Willmann 2001: 551). As far as its relation to the concept of legitimation is concerned, this interpretation is less puzzling as it seems to be compatible with an understanding of legitimation as justification by giving reasons. Nonetheless, we have to ask: In what sense can we call an expectation to be legitimate? At least the argumentative structure of the answer seems to be clear: if hope is interpreted as something we expect, then its legitimacy would stem from what is desired or positively expected in the future, from a state of affairs that is supposed to be good and valuable.

This leads to the second question concerning the meaning of the terms: What is legitimate? Or more precisely: How do we understand the term in our context? If we start with the help of etymology, then the meaning of the term legitimacy can be traced back to the Latin “legitimus” which is translated as “rightful” or “lawful”. Its original meaning played a role in the practices used to define the status of a child, especially a son, who was regarded as “legitimus” if he stemmed from a lawful relation of a father and mother, or more precisely: if he was born in the realm of a rightful institution, in this case: marriage. His status as a “legitimate” child was thus derived from a social institution constituting the order of society. Thus, in more general terms, for someone to be recognized as legitimate, his or her status had to be derived from an institution that is generally acknowledged as rightful. Of course, in case of doubt, specific acts of justification can be more or less convincing in concrete cases. Yet, the logic of justifying someone’s status as “legitimate” in a social or political order would imply a reference to the normative principles on which this order had been build, rather than to the prospects, effects or consequences one could expect under the circumstances given. Needless to say, the tales of kings and queens are not the only well-known experiences that disputes about whether or not someone’s status is legitimate were not always conducted with the aim of finding out whether it was rightful that he or she acquired the status in question. With the benefit of hindsight, there are numerous examples that sometimes disputes about this question were tacitly influenced by expectations and prospects about the possible effects of the answer.

As a result, experiences like these suggest that one should follow a critical approach to the study of what is “legitimate”, which reflects both normative and empirical aspects (Saretzki 2009). In terms of concept building, many political scientists would want make this explicit by introducing a distinction between legitimacy and legitimation. Following this distinction, the concept of *legitimacy* refers to the normative claim that a political order (or something that is derived from this order) ought to be respected as legitimate as a whole because it can normatively be justified as rightful. The concept of *legitimation* is interpreted as referring to factual belief-systems of political actors, elites and citizens, who currently or over longer periods of time think that a specific political order actually is legitimate and deserves to be accepted. The term legitimation is also used in studies of practices of justification by giving reasons, that is, attempts of political actors to actually justify the order (he or she represents) as rightful at a particular place and time. These practices can be observed particularly in situations when actors representing a political order are challenged by attempts to delegitimize the order as such or criticize the way the office-holders interpret their rights and duties and use of power or resources within a given order (Nullmeier et al. 2010).

In political science, the concepts of legitimacy and legitimation were originally and primarily related to the order of a political system or even of society as a whole. Yet within such general frames, it is possible to raise questions concerning the legitimacy and legitimation of the order of policy subsystems (e.g. a policy field such as technology policy), systems of advising and counselling policy-makers, systems of assessing technologies or systems of technologies.

## Why participation? Approaches to justify participation in TA and TP

As far as its *legitimation* is concerned, one can distinguish two basically different approaches to justify participation in technology assessment and technology policy. One is *normative* in a straightforward sense insofar as it refers to basic principles of normative theories of democracy: If and to the extent that processes of technization and technologization are major forces in a democratic society, then its citizens or their representatives should have a chance to participate in the assessment and the policy-making processes related to the technologies in question as a consequence of the democratic principle of self-government of free and equal citizens. As the technology assessment process is seen as providing the grounds for judgements of possible problems and options to solve them, TA is conceived of as part of the democratic opinion- and will-formation that precedes and predetermines the decision-making in the field of technology policy and thus is also subject to claims for democratic participation.

While this explicitly normative principle of justification can be and mostly is interpreted to include both technology assessment and technology policy as part of the deliberation, it allows for a great deal of latitude with regard to the *model of democracy* and the model of citizenship that serve as the normative system of reference. These models can vary considerably. A first and very often cited distinction had already been pointed out by James Carroll (1971: 647): In his definition, “participatory technology” refers to the inclusion of people in two different ways, namely either “directly” or “through agents under their control”. Thus, the idea can be interpreted with reference to a model of direct democracy or some form of participatory democracy on the one hand or established institutions of representative or parliamentarian democracy on the other hand. Further differences between other models of democracy (e.g. models of liberal, republican or deliberative democracy) may come into play (Saretzki 2002: 96–100, Biegelbauer and Hansen 2011). What is required in this explicitly normative approach of justification is a line of reasoning that explicates a reference to the basic democratic principle of self-government of free and equal citizens.

The other approach to justify participation in technology assessment and technology policy is *functional*: In this view, participation is justified as being instrumental in achieving certain goals and fulfilling certain functions that provide the basis of its legitimation. New technologies should be introduced (and old technologies should be evaluated) with broad public participation, so these arguments run, because participation fulfils certain functions more effectively and efficiently than other (non-participatory) ways of assessing technologies and policy-making in technology-related fields. Functional justifications can focus on informational, social, spatial or time-related dimensions of technization processes, of technology assessment or of technology policy.

In this functional line of reasoning, the *informational* dimension is most prominent and often seen as providing the grounds for other functional considerations. As far as TA is concerned, it provides the rationale for the whole enterprise. Seen the other way round, participation was justified as part of an ideal concept and methodology of TA. In the German discussion, participation is one of five basic elements of the concept of a “comprehensive TA” (Paschen and Petermann 1991: 26–30). Apart from such conceptual and methodological justifications, participation is sometimes presented as an instrument of information distribution or information collection, that is, it can help gaining access to the *“local”* knowledge of potential users or affected groups. As far as the *social* dimension is concerned, participation can be seen as an instrument of overcoming sceptic and hostile attitudes among certain groups in specific places or the citizenry at large, helping to create public understanding and acceptance. As for the *time* dimension, there are many calls for early participation of potentially interested, affected or engaged groups, often suggesting that such early inclusion, although costly in time (and money) upon first sight, might actually save time at the end of the day, if it helps avoiding conflicts usually leading to delays of the original schedule. Within a functional approach, various combinations of different dimensions are possible.

The conceptual reflections reveal that the juxtaposition of participation in “questions of technology” as being either “legitimate hope” or “mere illusion” presupposes a functional mode of justification. What about the other pole of the juxtaposition: “mere illusion”?

## Empirical analysis and evaluation of participatory processes in TA

In the light of the various participatory experiments in TA that have been analysed and evaluated by social scientists, it seems obvious that these scholars did not have illusions and did not write fiction. There is no denying the fact that there are practical experiences with participation in TA. What scholars have in mind when they create an alternative with one pole of the juxtaposition framed as “mere” or “grand illusion” can be translated into a concern with the scope and the meaning of “participation”. Recollecting that the diagnosis of James Carroll (1971) is already 40 years old, one might raise the question whether or not there is still a trend towards “participatory technology”.

What Nelkin and Pollak (1979: 55) found in their comparative study at the end of the 1970s can be extended to the following periods: participatory designs invented by governments as reforms “showed considerable *variations* with regard to their understanding of public participation and the role of technical and political information in the decision-making processes” (emphasis added, cf. for later studies in pTA, for example Joss and Bellucci 2002; Abels and Bora 2004; Hennen et al. 2004; Reber 2007; Felt and Fochler 2008).

Participatory projects in TA differed in terms of their topics, goals, their models of representation, modes of participation and communication, their structure and organization as well as their locations and time frames and, finally, the strategies of the actors involved. With regard to the often controversial dimension of *representation,* empirical studies showed designs focussing on expert groups (including only scientists or broader categories of professionals), stakeholder groups (including established interest groups, engaged activists or citizen groups), office-holders and political representatives from non-partisan institutions or partisan organizations (political parties or associations). Sometimes representation was constructed by open recruitment based on random samples or everyman participation. The modes of *participation* varied in terms of the range of rights and duties (information, hearing, initiative, vote), the concept of equality (equal access vs. equal opportunity), and the structure showed variations ranging from constant and continuous forms (centralized, e.g. conclave) to more differentiated patterns of participation (decentralized, e.g. by topic, social or spatial criteria). Empirical analysis also revealed remarkable variations with regard to different *contexts* of the projects like the context of initiation, the institutional context, the context of utilization and the broader societal and cultural context.

Although the general picture is one of heterogeneity, empirical studies and evaluations also pointed to some *trends*. The emergence of the projects could often be explained as a reaction to specific (“wicked”) problems and as a reaction to specific institutional legitimation problems. The timing suggested a certain correspondence with the overarching waves of democratization (or their decline). Some models like the consensus conference appeared to follow models of policy transfer and diffusion, indicating a sort of normalization and standardization in the field of participatory TA. According to some critics there are projects showing signs of being instrumentalized, while other critical observers saw more general trends in the direction of further professionalization, commercialization or industrialization.

*Evaluations* differed in terms of the underlying social perspectives of the roles involved (initiator, sponsor, coordinator, facilitator, organizer, participant, addressee, observer) and their points of reference, that is, whether the evaluation focussed on the process or the result (or both), more specifically, whether the preferred object of evaluation was the concept, structure, course, output, impact or even outcome. As far as the results are concerned, evaluations differed in terms of their levels of reference: Some remained within the typical range of TA, evaluating the information value of particular arguments, the comprehensiveness of all relevant arguments or the level of argumentative reflexivity and cooperation in the conceptualization of complex controversies (rational dissent). Already at the border of the domain of technology policy are evaluations that focus on questions like: Did the participants achieve a substantial consensus on policy proposals? And some evaluators would only give good credits if the project actually ended in the effective implementation of a consensus on policy proposals (implemented consensus). Finally, there were controversies with regard to the criteria of evaluation, which could be classified as referring either to the performance or to the legitimacy of the project (Saretzki 2011: 226–227). This leads to the question of legitimation problems of participation in TA.

## Legitimation problems of participatory processes in TA?

If we understand “problems” as being defined by a difference between an actual state and a state that ought to be achieved, then *legitimation problems* indicate that attempts to justify participation in a given case have not been entirely successful in the eyes of relevant groups among the sponsors, participants, organizers or observers of a participatory process. A rational reconstruction of the problems these (at least partly critical) groups have identified with regard to the legitimation of participation will have to make explicit reference to the approaches and criteria that have been introduced to justify it in the first place. In the light of such reconstructions, one can see that legitimation problems of participatory processes in technology assessment and technology policy vary considerably, and they do so not only with the two domains and the ways of their interrelation, but also with specific features of the participatory processes such as their concrete topic, mode of representation, timing, method of inquiry or procedure of deliberation and decision-making. If we ask whether or not participation is seen as problematic in technology assessment and technology policy-making and in what sense it is being evaluated as problematic, then we find that the answer depends also on the approaches and criteria that have (implicitly or explicitly) been used to legitimize or delegitimize the call for and specific design of participation.

A rational reconstruction of claims concerning legitimation problems of participatory processes in TA would try to interpret and reconstruct the reasoning of an affirmative or critical judgement in question and make its sometimes tacit (normative) assumptions explicit. If an explicitly normative approach based on principles of democratic legitimacy provided the legitimation for (a specific type of) participation, then legitimation problems would (in a rational reconstruction) have to be (re-)conceptualized by identifying a lack of compatibility, conflicts or even contradictions between the participatory procedures and practices observed and the principles and norms of democracy, with the concrete problem definition depending on the specific normative models of democracy and citizenship applied. If a functional approach based on criteria of performance provided the legitimation for (a specific type of) participation, then legitimation problems would be formulated by identifying a lack of the performance or even total failure in terms of the functions assigned or expected with regard to the information, social, spatial or time dimension.

In the debates about participation in TA, legitimation problems have been identified with regard to process and to results. In evaluations focussing on problems related to the process dimension (explicit or implicit) references to normative approaches of legitimation played an important role, whereas functional considerations were dominating when issues of performance were at stake. At least upon second sight, differences in evaluation did not pass uncontested, but lead to controversies. In *controversies* with regard to *process*, the focus was on questions of openness in the social (who had access?), temporal (enough time?) and substantial dimension (predetermination of results?), on issues of representativeness (who is representing whom, who should be represented, but is not?), on competences (rights and duties of participants), on fairness of the procedural design, on rules of decision-making (majority vote and if so, minority report?) and on behaviour of various actors involved. Controversies with regard to *results* focussed on questions of factual adequacy and comprehensiveness, problem-solving capacity of policy proposals, consensus building and acceptance generating capacity, binding force of recommendations and chances of implementation of policy proposals. Some of the controversies referred explicitly to the relation of the participatory procedure to its social, economic, political and cultural *context*. This is where issues concerning the *relation of TA and technology policy* play an important role. Critics raised questions concerning issues of (mis-)representation, (limited) rights of participants, unequal resource distribution among different groups of participants leading to “participation overload” or even “overkill” of some groups (Spangenberg 1993). Some critics saw signs that groups involved were trying to instrumentalize a participatory project, which could come “from above” using participation for external purposes of producing legitimation and acceptance or fostering cooptation, adjournment or displacement. Of course, participatory procedures are also open to attempts of instrumentalization “from below”, aiming not only at delegitimation of certain established positions, but also at mobilization, protest or postponement of a technological project. Moreover, the need to provide facilitation and professional coordination of sometimes complex participatory designs leads to questions concerning possible conflicts of interest of facilitators and mediators and possible consequences of intensive participation in professionally managed consultation projects for the capabilities of citizen groups for their self-organization (Saretzki 2008: 48–51; Felt and Fochler 2008; Fischer 2009: 99–102; Hoppe 2011: 215–241; van Oudheusden 2011; Guston 2011).

## Legitimation problems of policy analysts in participatory TA-processes?

As long as TA was functionally presented as a scientific activity like research and institutionally located as if it was simply a part of the scientific system, TA-analysts could interpret and legitimate their own role and activities with the help of traditional images of a “neutral” or “objective technician” based on classical models of positivistic science. To the extent that TA is now institutionally situated between the scientific and the political system, oriented also towards a broader public and is no longer perceived as part of the scientific system, the classical legitimation with recourse to the old role as scientific expert is put into question. Moreover, to the extent that people become aware of the epistemic limits of positivistic science for problem-solving and policy-oriented analysis, the role of the “objective technician” is also internally cast into doubt. On the other hand, situating oneself in the institutional space as part of the political system and interpreting one’s own professional analyses simply as nothing but another kind of political advocacy in a field of pluralistic interest group competition would also miss the tasks of TA. And it would create other problems of legitimation regarding the role of a specific “knowledge holder” communicating and mediating between science, politics and the public. These legitimation problems are increasing, if and to the extent that participation becomes an element or even a basic feature in the TA-process itself. Thus, given these new conditions, TA-analysts cannot and should not try to refer to normative systems of either science or politics. Rather, they face the challenge of developing a *TA*-*specific set of principles and norms* which can serve as a system of reference for *legitimating their own new roles*, especially in the context of participatory procedures in TA.

For this reorientation, a look at the debates in *policy analysis* may be helpful. TA has already been conceptualized as policy analysis (Reber 2007) or at least as entailing some kind of policy analysis (as a final step based on the assessment of technology impacts and possible fields of social conflict, cf. the classical TA concept of Paschen and Petermann 1991). Moreover, experienced practitioners of TA have already very early pointed to the “insight that TA analyses are to be understood as argumentative processes” (Paschen et al. 1978: 58, my translation). Remembering such insights, TA may now under new conditions of extended participation learn especially from new ideas that are associated with “the argumentative turn in policy analysis and planning” (Fischer and Forester 1993). The take-home messages of these calls for reorientation can be summarized as follows: Rethink policy analysis (in TA) and its relations to science and politics! That means on the one hand: Rethink the model of science! Leave the focus on data and trust in abstract economic methods, realize the uncertainty and ambiguity of scientific knowledge and turn to a post-positivist concept of science. On the other hand: Rethink the model of politics! Overcome decisionist and technocratic models of political decision-making, change orientation from elites to all sorts of interested, affected or engaged actors and diverse publics, turn to an explicitly democratic concept of politics. Moreover: Rethink your own role as policy analyst! Do not uphold the image of “analyst as objective technician”, accept the ambiguity of the roles a policy analyst has to fulfil (in TA). Finally: Rethink the model of counsel and advice! Realize the importance of language and argumentation, change your paradigm from “analysis as science” towards “analysis as argument”! (Saretzki 2012).

While the paradigm of policy analysis as science implied the role of an “objective technician” focussed on data collection and analysis, the functions and roles of policy analysts in participatory TA-projects are much more diverse and complex. On the one hand, the concept of policy analysis as argument implies that analysts should work on the tasks of cognitive differentiation and knowledge integration of impacts and options. These tasks will confront them with the analytical challenge of operating as an interpreter, translator and producer of arguments, working on the boundaries of various scientific disciplines, the local knowledge and the life-world of other participants. On the other hand, the concept of policy analysis as participatory procedure requires a procedural structuring of the analytic-deliberative process and communication with the public. This task will confront the TA-analyst with the challenge of working as designer, facilitator and moderator of the participatory process (Saretzki 2005: 363–367).

## Summary and conclusion

The idea of a “participatory technology” was invented as a response to legitimation problems of technology in society and of technology policy in a democracy. Participation in technology assessment turned from a normative claim to a working practice. Yet, the “if” and “how” of participation are still contested issues. While participation was originally meant to solve legitimation problems of technology and technology policy, the participatory problem-solving strategies also led to specific new legitimation problems of their design and implementation. TA and technology policy are still two different, but strongly interrelated domains. To understand their legitimation problems in concrete cases, the specific ways of their interrelation in concrete contexts need to be taken into account. From the perspective of a political scientist focussing on the policy process, TA is not conceptualized as a set of instruments from a tool box or as a social technology, but as an analytic-deliberative process mediating between the functionally and institutionally differentiated systems of science and politics and the public.

Empirical studies of participatory processes in TA and TP show variations in a number of different analytical dimensions. Evaluations are accompanied by controversies over different aspects of legitimacy and performance. Evaluating participation in TA-processes within the juxtaposition of “legitimate hope versus mere illusion” presupposes a functional justification. In the context of functional justifications, problems of legitimation are better understood and should be discussed as problems of performance. Far reaching, but disappointed hopes about expected consequences of participation cannot provide the normative grounds to deny its legitimacy. They can and should induce efforts to improve the performance of participatory projects with regard to the functions assigned.

New tasks in participatory TA-processes create new roles for policy analysts with new “threads and temptations of power” (Lasswell 1974: 177). As a result, policy analysts working in participatory processes cannot simply take recourse to the justifying principles of either science or politics. They are confronted with new challenges for the legitimation of their professional activities in TA which require a multidimensional, self-reflective and self-critical approach (Saretzki 2008).

## References

[CR1] Abels G, Bora A (2004) Demokratische Technikbewertung, transcript, Bielefeld

[CR2] Biegelbauer P, Hansen J (2011). Democratic theory and citizen participation: democracy models in the evaluation of public participation in science and technology. Sci Public Policy.

[CR3] Carroll J (1971). Participatory technology. Citizen participation in the public development, use, and regulation of technology is examined. Science.

[CR4] Felt U, Fochler M (2008). The bottom-up meanings of the concept of public participation in science and technology. Sci Public Policy.

[CR5] Fischer F (2009). Democracy & expertise. Reorienting policy inquiry.

[CR6] Fischer F, Forester J (1993). The argumentative turn in policy analysis and planning.

[CR7] Guston DF (2011). Participating despite questions: towards a more confident participatory technology assessment. Sci Eng Ethics.

[CR8] Hennen L, Petermann T, Scherz C (2004). Partizipative Technikfolgen-Abschätzung und Parlamentarische Politikberatung.

[CR9] Hoppe R (2011). The governance of problems: puzzling, powering and participation.

[CR10] Hornby AS (1972). Advanced learners dictionary of current English.

[CR11] Joss S, Bellucci S (2002). Participatory technology assessment. European perspectives.

[CR12] Lasswell H (1974). Some perplexities of policy theory. Soc Res.

[CR13] Nelkin D, Pollak M (1979). Public participation in technological decisions: reality or grand illusion?. Technol Rev (August/September).

[CR14] Nullmeier Frank (2010). Prekäre Legitimitäten. Rechtfertigung von Herrschaft in der postnationalen Konstellation.

[CR15] Paschen H, Petermann T, Petermann H (1991). Technikfolgen-Abschätzung—ein strategisches Rahmenkonzept für die Analyse und Bewertung von Techniken. Technikfolgen-Abschätzung als Technikforschung und Politikberatung.

[CR16] Paschen H, Gresser C, Conrad F (1978). Technology assessment: Technologiefolgenabschätzung.

[CR17] Reber B, Fischer F, Miller GM, Sidney MS (2007). Technology assessment as policy analysis: from expert advice to participatory approaches. Handbook of public policy analysis. Theory, politics, and methods.

[CR18] Saretzki T, Heinelt H, Getimis P, Kafkalas G, Smith R, Swyngedouw E (2002). Technological governance—technological citizenship?. Participatory governance in multi-level context: concepts and experience.

[CR19] Saretzki T, Mensch K, Schmidt JC (2003). Gesellschaftliche Partizipation an Technisierungsprozessen: Techniksteuerung von unten?. Technik und Demokratie. Zwischen Expertokratie, Parlament und Bürgerbeteiligung.

[CR20] Saretzki T, Bogner A, Torgersen H (2005). Welches Wissen—wessen Entscheidung? Kontroverse Expertise im Spannungsfeld von Wissenschaft, Öffentlichkeit und Politik. Wozu Experten? Ambivalenzen der Beziehung von Wissenschaft und Politik.

[CR21] Saretzki T, Janning F, Toens K (2008). Policy-Analyse, Demokratie und Deliberation: Theorieentwicklung und Forschungsperspektiven der „Policy Sciences of Democracy“. Die Zukunft der Policy-Forschung. Theorien, Methoden, Anwendungen.

[CR22] Saretzki T (2009). Habermas and critical policy studies: legitimation, judgement, and participation. Crit Policy Stud.

[CR23] Saretzki T, Kauffmann C, Sigwart HJ (2011). Biopolitik in diskursiven Designs: Empirische Analysen und politiktheoretische Implikationen. Biopolitik im liberalen Staat.

[CR24] Saretzki T, Egner B (2012). The "argumentative turn" revisited: Demokratisierung von Policy-Analysen in partizipativen Projekten und diskursiven Designs?. Regieren.

[CR25] Spangenberg J (1993) Participation overkill. In: Die Tageszeitung. 10 April 1993, p 6

[CR26] Stern PC, Fineberg H (1996). Understanding risk. Informing decisions in a democratic society.

[CR27] Van Oudheusden M (2011). Questioning ‘participation': a critical appraisal of its conceptualization in a Flemish participatory technology assessment. Sci Eng Ethics.

[CR28] Willmann H (2001). Langenscheidts Großwörterbuch Englisch Muret-Sanders.

[CR29] Winner L (1980). Do artifacts have politics?. Daedalus.

